# Developmental stage-specific triterpenoid saponin accumulations in *Ardisia crenata* rhizosphere and its influence on rhizosphere microbial communities

**DOI:** 10.5511/plantbiotechnology.25.0504a

**Published:** 2025-09-25

**Authors:** Naoto Nakamura, Akifumi Sugiyama

**Affiliations:** 1 Laboratory of Plant Gene Expression, Research Institute for Sustainable Humanosphere, Kyoto University, Uji, Kyoto 611-0011, Japan

**Keywords:** ardisiacrispin, DNA metabarcoding, plant-microbe interaction, plant specialized metabolites, root exudation

## Abstract

Plant-specialized metabolites (PSMs) act as candidate drivers of rhizosphere microbiome assembly by recruiting specific microbial taxa. The resulting PSM–microbe interactions influence the host-plant fitness and population dynamics, ultimately impacting the aboveground biodiversity. Although saponins are widely distributed PSMs in the angiosperms, their dynamics and impact on soil microbiomes in a natural ecosystem remain unclear. Here, we investigated the ecological role of a triterpenoid saponin, ardisiacrispin, synthesized by the shade-tolerant shrub *Ardisia crenata* (Primulaceae), in a forest ecosystem. First, we quantified the saponin concentrations in both the roots and rhizosphere soils of *A. crenata* at two different developmental stages (i.e., seedling and adult). Next, we assessed how saponin treatment alters the microbial communities in forest soil. Finally, we integrated 16S rRNA and the internal transcribed spacer region sequencing data from the field-collected *A. crenata* rhizosphere with the results from *in vitro* saponin-treatment experiments to determine whether saponins selectively enrich or deplete specific microbial taxa. We found that the rhizosphere saponin content primarily varies with the developmental stages of *A. crenata*, with higher saponin concentrations in adults than in seedlings. The saponin-treatment experiments revealed that ardisiacrispins modify the soil microbial diversity and community structure in accordance with their concentration. Moreover, several microbial taxa were consistently enriched or depleted in the saponin-treated soil, which mirrors the shifts observed from seedling to adult rhizospheres. Thus, ardisiacrispin can mediate rhizosphere microbial community assembly in a natural ecosystem. Our findings highlight the importance of the developmental stage-specific accumulation of saponins in the rhizosphere for plant-microbe interactions.

## Introduction

Rhizosphere microbial communities, which are shaped by interactions among microbes and between microbes and host plants, play multifaceted roles in plant growth and health by influencing nutrient acquisition, stress tolerance, and disease suppression (Berendsen et al. 2012; Finkel et al. 2017; Hacquard et al. 2015). These assemblages are highly diverse and have such beneficial functions, but they are simultaneously subjected to selective pressures from plant-derived exudates (Hassani et al. 2018; Oburger and Jones 2018; Yu et al. 2019). Indeed, plant root exudation regulates the accumulation and establishment of rhizosphere microbes (Pantigoso et al. 2022), and these interactions can affect not only the host–plant fitness but also species diversity and population dynamics at the community level, which ultimately exert broader impacts on the entire ecosystem (Poppeliers et al. 2023; Trivedi et al. 2020).

Plant specialized metabolites (PSMs) are diverse secondary metabolites synthesized and secreted by plants that play important roles in shaping species-specific rhizosphere microbial communities (Massalha et al. 2017; Pang et al. 2021). Advances in sequencing and analytical technology have revealed that various plant species exudate distinct PSMs through their roots, such as saponin, coumarin, and flavonoids, each exerting unique effects on the structure and function of soil microbial communities (Herz et al. 2018; Hu et al. 2018; Okutani et al. 2020; Shimasaki et al. 2021; Yuan et al. 2018; Zhalnina et al. 2018). For instance, soyasaponin Bb in rhizosphere soil accumulates *Novosphingobium*, while isoflavonoids in rhizosphere soil accumulate Comamonadaceae (Sugiyama 2023). Although these findings suggest that multiple PSMs broadly affect microbial community composition and interactions beyond agricultural systems, previous studies have predominantly examined PSMs from model or crop species in agricultural soils, leaving their roles in natural ecosystems less explored. Because the effect of PSMs on the rhizosphere soil microbiome depends on the soil type (Cadot et al. 2021; Sasse et al. 2018), a deeper understanding of the ecological importance of PSM-rhizosphere soil interactions requires further research that extends beyond agricultural contexts, for instance, a research that includes forest soils and their associated plant species.

Saponins are widely distributed PSMs in angiosperms and exhibit diverse biological and pharmacological activities, including antibacterial, antifungal, and cytotoxic activities (Sparg et al. 2004). Particularly, studies on soybean and ginseng have demonstrated that saponins accumulated in the roots can be released into the rhizosphere, thereby influencing the structure and function of rhizosphere microbial communities through the enrichment of specific microbial taxa (Luo et al. 2020; Tsuno et al. 2018). For instance, Fujimatsu et al. (2020) studied the exudation rate of soyasaponin Bb varies with plant developmental stages under field conditions and found that soyasaponin facilitates the enrichment of *Novosphingobium* in the rhizosphere. Similarly, Nakayasu et al. (2021b) treated field soil with saponins derived from multiple plant species *in vitro*. They found that certain bacterial genera, including *Phenylobacterium* and *Sphingobium*, were selectively enriched across diverse saponin types. Although these studies highlight the potential of saponins in regulating rhizosphere microbial dynamics in both field and laboratory contexts, very little is known about how saponin exudation changes with plant growth in the natural ecosystems or how such changes influence the rhizosphere microbial communities.

**Figure 1:**
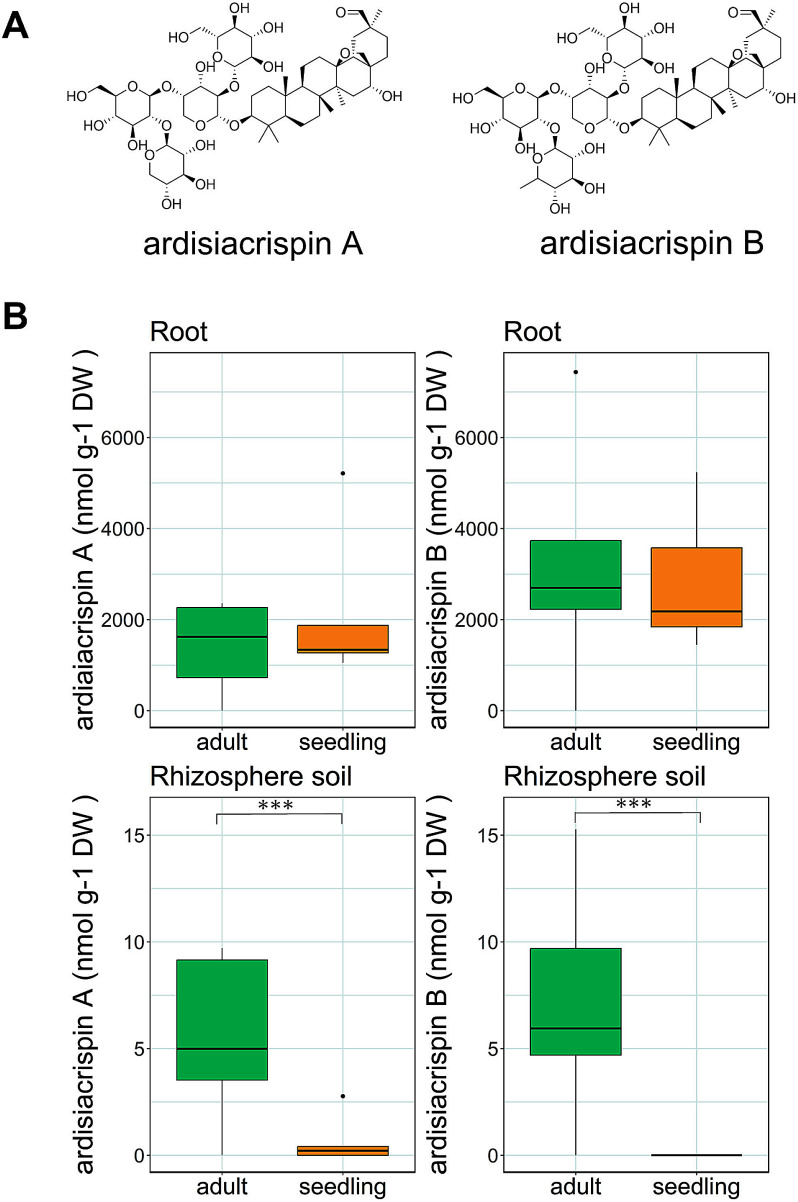
Figure 1. Chemical structures of ardisiacrispin A and ardisiacrispin B, with difference in the terminal sugar moieties (A). Concentrations of ardisiacrispin A and ardisiacrispin B in roots and rhizosphere soil of *A. crenata* at two developmental stages (B). In the rhizosphere soil of seedlings, ardisiacrispin A was undetected in three out of five samples, while ardisiacrispin B was undetected in all samples. The concentration of ardisiacrispin in samples judged as “undetected” is estimated to be below 0.1 nmol g^−1^. Significant difference among invasion statuses as indicated by asterisks, determined by the Wilcoxson test (*** *p*<0.001).

In this study, we focused on *Ardisia crenata*, a shade-tolerant shrub native to East Asia that has invaded the forest floor in the southeastern United States (Dozier 1999; Kitajima et al. 2006; Nakamura et al. 2023). The roots of *A. crenata* accumulate high contents of triterpenoid saponins, which are commonly used for medicinal purposes (Jia et al. 1994; Tian-Liang et al. 2024). Ardisiacrispins are a predominant triterpenoid saponin group produced by *A. crenata*, among which ardisiacrispin A and ardisiacrispin B are particularly abundant (Ma et al. 2015). Ardisiacrispin A and ardisiacrispin B are a pair of saponins that share the same aglycone structure but differ in their terminal sugar, which is either a xylopyranosyl or a rhamnopyranosyl moiety, respectively (Figure 1A). Ardisiacrispin A exhibits cytotoxic and uterotonic activities (Podolak et al. 2021), whereas ardisiacrispin B demonstrates cytotoxic and anti-inflammatory effects (Mbaveng et al. 2018; Zhou et al. 2023). However, it remains unclear whether these saponins are exuded into forest soils via the *A. crenata* roots and how they might shape the rhizosphere microbial community. Understanding the ecological impact of ardisiacrispins is expected to shed light on the importance of secondary metabolites in plant-microbial interactions beyond their role in the ecological processes through which invasive species alter the soil environments.

To explore the ecological effect of saponins on the rhizosphere microbiome in natural ecosystems, we examined the concentration of saponins in both the roots and the rhizosphere of *A. crenata* at two different developmental stages (i.e., seedling and adult) as well as evaluated how saponin treatment alters the microbial communities in the forest soil. Finally, we integrated field observations with *in vitro* saponin treatment experiments to examine whether saponins in the *A. crenata* rhizosphere selectively enrich or deplete specific microbial taxa.

## Materials and methods

### Study site and sampling

*A. crenata* sampling was conducted at the Kamigamo Experimental Station, Kyoto, Japan (35.07°N, 135.76°E) in May 2024. This forest canopy is dominated by *Chamaecyparis spp.*, with patches of *A. crenata* present in the understory, covering approximately 5% of the forest floor. A 1×10-m transect was established and divided into ten 1×1-m plots. From these, five sampling plots were selected at least 1-m apart from each other. Within each sampling plot, one adult individual of *A. crenata* (≥20 cm in height) and one seedling individual (≤10 cm in height) were selected as closely as possible to each other. It takes at least three years to reach adult size and estimated to be less than one year old since germination (N. Nakamura, personal observation, Supplementary Figure S1). *A. crenata* become reproductive when they grows to approximately 20 cm in height (Kitajima et al. 2006). The roots and rhizosphere soil were collected from selected individuals, and bulk soil was sampled from the plot away from the selected individuals. All samples were transported to the laboratory in a cool container (0–10°C) within 1 h. The root samples were sonicated to remove the attached soil. Subsequently, they were ground under liquid nitrogen with mortar and pestle, and stored at −80°C for DNA analysis and root exudate extraction. The rhizosphere soil samples were then separated from the roots by brushing off the attached soil and passing through a 2-mm sieve to remove the larger debris. These samples were then stored at −20°C for subsequent DNA analysis and root exudate extraction.

### Chemicals

Ardisiacrispin A and ardisiacrispin B standards were purchased from GlpBio (Montclair, California, USA) and used as reference compounds to estimate the saponin content in *A. crenata* samples as well as standard compounds for the saponin treatment experiments. Other chemicals were purchased from FUJIFILM Wako Pure Chemical Industries Ltd. (Osaka, Japan) or Nacalai Tesque Inc. (Kyoto, Japan) unless otherwise stated.

### The analysis of ardisiacrispins in the rhizosphere soil and the roots of *A. crenata*

To extract saponins from the root samples, 300 µl of methanol was added to each sample and subjected to vortexing, sonication for 15 min, and centrifugation at 10,000×g for 5 min. The supernatant was collected. This extraction process was repeated two times. To extract the saponins from the rhizosphere soil, 4 ml of methanol was added to each sample, followed by vortex mixing, incubation for 40 min at 50°C, and centrifugation at 5,000×g for 5 min. The supernatant was collected and the extraction process was repeated thrice. The obtained supernatant was dried, and the residue was dissolved in methanol. All prepared samples were filtered through a 0.45-µm Minisart RC4 filter (Sartorius, Göttingen, Germany) before analysis by liquid chromatography-mass spectrometry (LC-MS). The concentrations of ardisiacrispin A and ardisiacrispin B were analyzed using the Acquity ultra-high-performance LC (UPLC) H-Class/Xevo TQD system (Waters). For each sample, 2 µl of the sample solution was injected into Acquity UPLC BEH C18 columns (1.7 µm, 2.1×50 mm; Waters). The column oven temperature was set to 40°C, and the mobile phases were composed of water with 0.1% (v/v) formic acid (solvent A) and acetonitrile with 0.1% (v/v) formic acid (solvent B). The flow rate was set to 0.2 ml min^−1^. Mass spectrometry was performed in the negative ionization mode under the following conditions: cone voltage, 50 V; desolvation gas temperature, 400°C; and nitrogen gas flow rates for the nebulizer and desolvation set to 50 l h^−1^ and 800 l h^−1^, respectively. The acquired data were analyzed using MassLynx v. 4.1 software (Waters). Ardisiacrispin A and ardisiacrispin B were quantified based on the peak areas in comparison with those in the calibration curves from the reference compounds.

### Saponin treatment experiments

To examine the effect of ardisiacrispins on the soil microbial communities *in vitro*, the soil samples were treated with ardisiacrispin A and ardisiacrispin B and incubated under controlled conditions. Two distinct soil types were used in this experiment to assess whether the effects of saponins on the microbial communities differed among the different soil types. The first soil type was forest soil (Kamigamo), which was collected as bulk soil from the same site where *A. crenata* was sampled at the Kamigamo Experimental Site, Kyoto, Japan. The second soil type was field soil (Kameoka), collected from the Kyoto University of Advanced Science, Kameoka, Japan (34.9938°N, 135.5514°E). The soil samples were air-dried and passed through a 2-mm sieve to remove the plant residues.

Saponin treatments were conducted as per a previously described protocol (Nakayasu et al. 2021b; Sugiyama 2023), albeit with minor modifications. Briefly, ardisiacrispin A and ardisiacrispin B were dissolved in methanol and dried in 5-ml tubes before application to soil. Five treatment groups were established for each soil type, as follows: (i) Control, where soils were treated with sterile water only; (ii) low treatment (50 nmol) of ardisiacrispin A; (iii) low treatment (50 nmol) of ardisiacrispin B; (iv) high treatment (200 nmol) of ardisiacrispin A; (v) high treatment (200 nmol) of ardisiacrispin B. The 50 nmol and 200 nmol saponin-treatment concentrations were selected with reference to previously reported rhizosphere saponin exudation concentrations in tomato and soybean (Nakayasu et al. 2021a; Tsuno et al. 2018). For each treatment, 2 g of soil and 600 µl of distilled water were added into 5-ml tubes (*n*=4/treatment). The tubes were sealed, vortexed, and incubated at 28°C in the dark. Every 3 days over a 15-day incubation period, each soil sample was transferred into a new tube containing the respective saponin compounds. The repeated addition of metabolites (e.g., transferring to a new tube) every three days, along with the total incubation period of 15 days, was based on our previous studies (Nakayasu et al. 2021b; Okutani et al. 2020), which demonstrated that this duration is sufficient for plant-derived specialized metabolites to alter soil microbial communities. During the course of the experiment, this resulted in a total saponin addition of 250 nmol for the low treatment and 1,000 nmol for the high treatment protocol. Following incubation, all samples were stored at −20°C until DNA extraction. Furthermore, to examine the extent to which ardisiacrispin A, ardisiacrispin B, and their aglycones remained in the soil, their contents in the saponin-treated samples were analyzed by LC-MS using the same methods described above.

### DNA extraction and amplicon sequencing

DNA was extracted from 0.25 g of each soil sample using the DNeasy PowerSoil Kit (QIAGEN K.K., Tokyo, Japan), as per the manufacturer’s protocol. We employed a two-step PCR approach for the library preparation for Illumina MiSeq sequencing. The bacterial 16S rRNA and fungal internal transcribed space (ITS) regions were PCR-amplified following the protocol detailed elsewhere (Toju et al. 2019) with some modifications. Briefly, the primer set 515f/806rB (515f, Caporaso et al. 2011; 806rB, Apprill et al. 2015) was used for the bacterial 16S rRNA region, and the primer set ITS4/ITS7 (ITS4, Ihrmark et al. 2012; ITS7, White et al. 1990) was used for the fungal ITS region. Both the primer sets were fused with the Illumina sequencing primer region. The first PCR was performed using KOD FX Neo (TOYOBO, Osaka, Japan) under the following conditions: an initial denaturation at 98°C for 120 s, followed by 20 cycles (for 16S rRNA) or 30 cycles (for the fungal ITS region) of 98°C for 10 s, 50°C for 30 s, and 68°C for 30 s. The PCR products were purified with the AMPureXP Kit (Beckman Colter, Danvers, MA, USA) to remove the primer dimers. The ratio of the AMPureXP reagent to each of the first PCR products was set to 0.5 (v/v). A second round of PCR (second PCR) amplification for the attachment of the Illumina MiSeq adapters was performed with the purified DNA as a template using KOD FX Neo and primers provided by FASMAC Co., Ltd. (Kanagawa, Japan). PCR cycling was conducted at 94°C for 2 min and 10 cycles of 98°C for 10 s, 59°C for 30 s, and 68°C for 30 s. The PCR products were purified with the AMPureXP Kit and quantified using the Qubit 2.0 Fluorometer (Thermo Fisher Scientific, Waltham, MA, USA). The ratio of the AMPureXP reagent to the second PCR product was set to 0.5 (v/v). The purified DNA was mixed in equal amounts and used for 2×250-bp paired-end sequencing with the Illumina MiSeq by FASMAC Co., Ltd. The 16S rRNA and ITS amplicon datasets supporting the results of this study are deposited under the accession BioProject PRJNA1247026 and are also available from the corresponding author upon reasonable request.

### Bioinformatics

The Illumina adapters were removed with Cutadapt4.4 (Martin 2011). The sequencing reads was subsequently processed with the package “DADA2” v.1.18.0 of R version 4.3.1 (R Core Team 2022) to remove low-quality sequences with a quality score of <20 and chimeras (function “FilterTrimming” and “removeBimeraDenovo”, respectively). The molecular identification of the obtained amplicon sequence variants (ASVs) was performed based on the naive Bayesian classifier method (Wang et al. 2007) with the SILVA v.132 databases for 16S rRNA and with the fungal UNITE database 9.0 release (Abarenkov et al. 2024) (function “assignTaxonomy”, DADA2). The obtained ASVs were clustered into operational taxonomic units (OTUs) at 97% similarity with “Vsearch” (Rognes et al. 2016) for both bacteria and fungi. To generate the final OTU table, we removed the nonbacterial and nonfungal OTUs. In total, 14,426 bacterial OTUs (3,360,451 reads) and 9,606 fungal OTUs (2,726,865 reads) were retained. The dataset was rarefied at 25,000 reads and 17,785 reads for bacteria and fungi, respectively, with the “rrarefy” function of the “vegan” package (Oksanen et al. 2022) of the R software. The samples that yielded less than these read numbers were discarded.

### Statistical analysis

To evaluate species diversity (α-diversity), we calculated the Shannon diversity of microbial communities as the exponent of Shannon entropy (i.e., exp(−∑*P_i_* ln(*P_i_*)); where *P_i_* is the proportional abundance of species *i*, Shannon 1948) calculated from the R package “vegan”. Significant differences in Shannon diversity among the soil samples were assessed using the nonparametric Wilcoxon test. To assess the differences in the microbial composition among the soil samples, permutational multivariate analysis of variance (PerMANOVA) was performed using the “adonis2” function in the “vegan” package in the R software, based on the Bray–Curtis distance values (9,999 permutations; Anderson 2006). Non-metric multidimensional scaling (NMDS) based on the Bray–Curtis dissimilarity was also conducted to visualize the differences in the microbial community structures among each sample using “vegan” and “ggplot2” (Wickham 2016).

To detect the microbial genera characteristics of the rhizosphere soil of *A. crenata* adult plants and seedlings, the linear discriminant analysis effect size (LefSe; Segata et al. 2011) algorithm was conducted. LefSe analysis was performed with an adjusted *p*-value threshold of <0.05 in the Kruskal–Wallis rank-sum test and a linear discriminant analysis score of >2.5 to identify statistically significant differences. Similarly, to evaluate the effects of the saponin treatment on the soil microbiome, LefSe analysis was conducted between the control and the 50-nmol saponin treatment, as well as between the control and the 200-nmol saponin treatment, without distinguishing the type of saponin. Taxa enriched or depleted in response to saponin treatment at either concentration were then merged and collectively defined as “saponin-enriched” or “saponin-depleted” taxa. To assess whether the effects of saponins on the microbial communities differed among the soil types, “saponin-enriched” and “saponin-depleted” taxa were compared between the forest and field soil samples. To infer the effect of ardisiacrispins on the soil microbiome under natural conditions, microbial genera that increased or decreased between the adult and seedling rhizospheres were compared with the saponin-enriched and saponin-depleted taxa in the saponin-treatment experiment of forest soil. Venn diagrams were generated using the VennDiagram package (Chen 2022) in R software.

## Results

### Saponin Content in the Root and Rhizosphere Soil of *A. crenata*

To examine the concentration of saponins in both the roots and rhizosphere of *A. crenata* at the two developmental stages, we conducted quantitative analysis using LC-MS. The roots of both the adult and seedling *A. crenata* contained ardisiacrispin A and ardisiacrispin B (Figure 1B). No significant difference was noted in the content of ardisiacrispin A and ardisiacrispin B in the roots between the adult and seedling. Meanwhile, the saponin content in the rhizosphere soil was higher in adults than in seedlings (Wilcoxon test, *p*<0.001). Ardisiacrispin A and B were nearly undetectable in the rhizosphere soil of seedlings; only two out of five samples contained ardisiacrispin A, while ardisiacrispin B was undetected in all samples. These results indicated that, although ardisiacrispins are produced at both developmental stages, their exudation into the rhizosphere appears to occur only in the adult stage.

### Microbial communities in the rhizosphere soil and bulk soil of *A. crenata*

**Figure 2:**
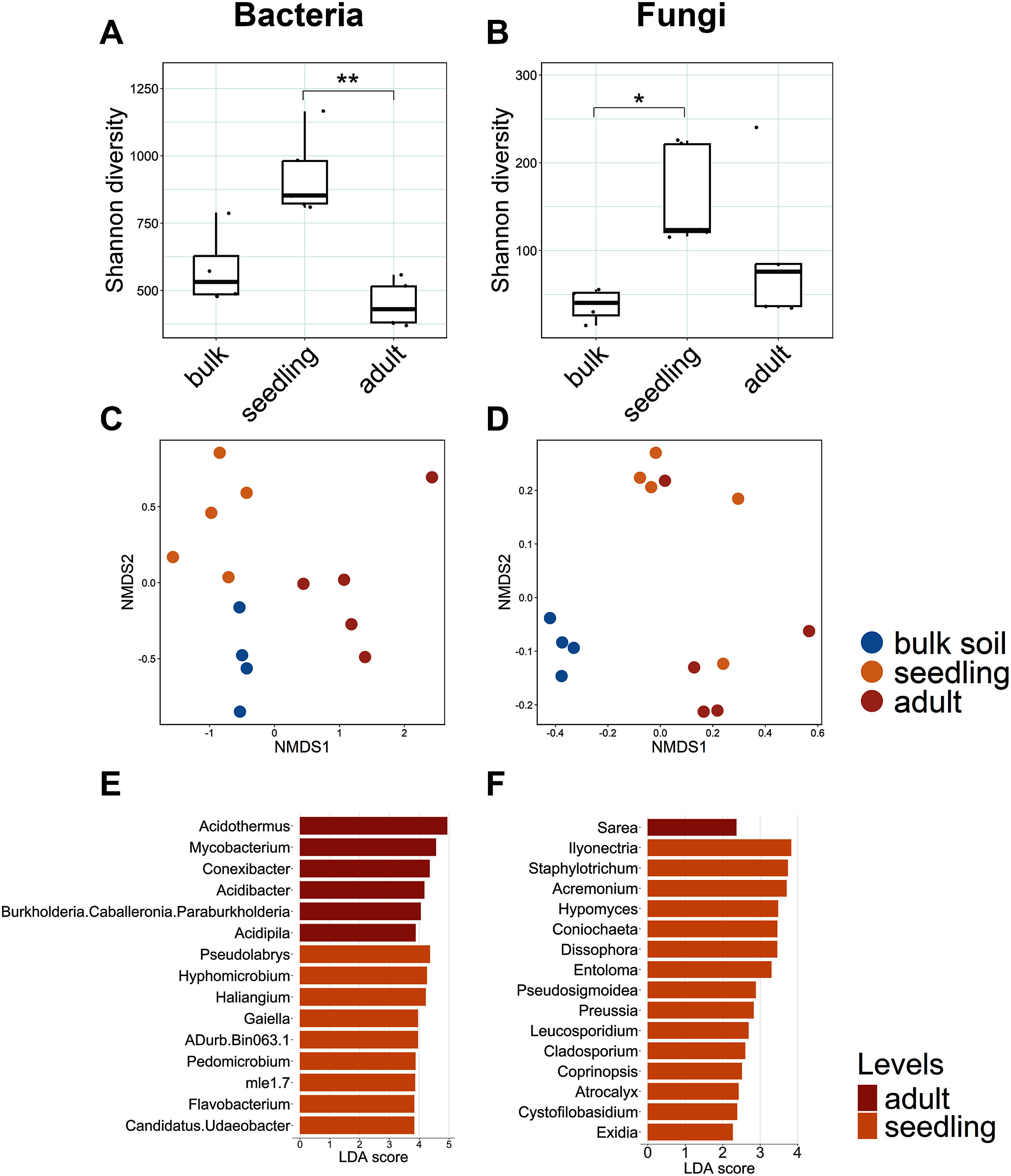
Figure 2. Shannon diversity of bacterial (A) and fungal (B) communities at bulk soil, seedling rhizosphere and adult rhizosphere. Significant difference was indicated by asterisks, determined by the Wilcoxon test (* *p*<0.05, ** *p*<0.01, *** *p*<0.001). Nonmetric multidimensional scaling (NMDS) plots based on Bray–Curtis distances of bacterial (C) and fungal (D) communities in three soil types of *A. crenata*: bulk soil (blue), rhizosphere soil of seedling (reddish brown) and rhizosphere soil of adult (red). Lefse analysis between adult and seedling rhizosphere for bacteria (E) and fungi (F) with top 15 genera were shown.

To investigate the microbial community associated with the *A. crenata* rhizosphere, we conducted 16S rRNA and ITS reason amplicon sequence analysis with three types of soil (i.e., adult rhizosphere, seedling rhizosphere, and bulk soil). For the bacterial communities, Shannon diversity was significantly higher in the rhizosphere of seedlings compared with that of adults (Wilcoxon test, *p*=0.003, Figure 2A). In contrast, the Shannon diversity of the rhizosphere of adults was lower than that in the bulk soil. NMDS analysis indicated that bacterial community structures in the rhizosphere differed between adults and seedlings (PerMANOVA, adjusted *p*=0.024), and both were distinct from those in the bulk soil (Figure 2C; Supplementary Table S1). LEfSe analysis at the genus level revealed that *Acidothermus*, *Mycobacterium*, and *Conexibacter* were characteristics of the adult rhizosphere, while *Pseudolabrys* and *Hyphomicrobium* were enriched in the seedling rhizosphere (Figure 2E).

For the fungal communities, Shannon diversity was significantly higher in the rhizosphere of seedlings than in that of the bulk soil (Wilcoxon test, *p*=0.013, Figure 2B). The Shannon diversity of the adult rhizosphere was not significantly different; it tended to be lower than that in seedlings. NMDS analysis indicated that the fungal community structures in the rhizosphere did not significantly differ between the adults and seedlings, but both were distinct from those in the bulk soil (Figure 2D, Supplementary Table S1). LEfSe analysis at the genus level revealed that *Sarea* was a characteristic of the adult rhizosphere, while *Ilyonectria* and *Staphylotrichum* were enriched in the seedling rhizosphere (Figure 2F). These results suggested a clear difference in the bacterial and fungal microbiomes among the three types of soil tested (i.e., adult rhizosphere, seedling rhizosphere, and bulk soil).

### Effect of ardisiacrispin treatment on the microbial communities in forest soil

**Figure 3:**
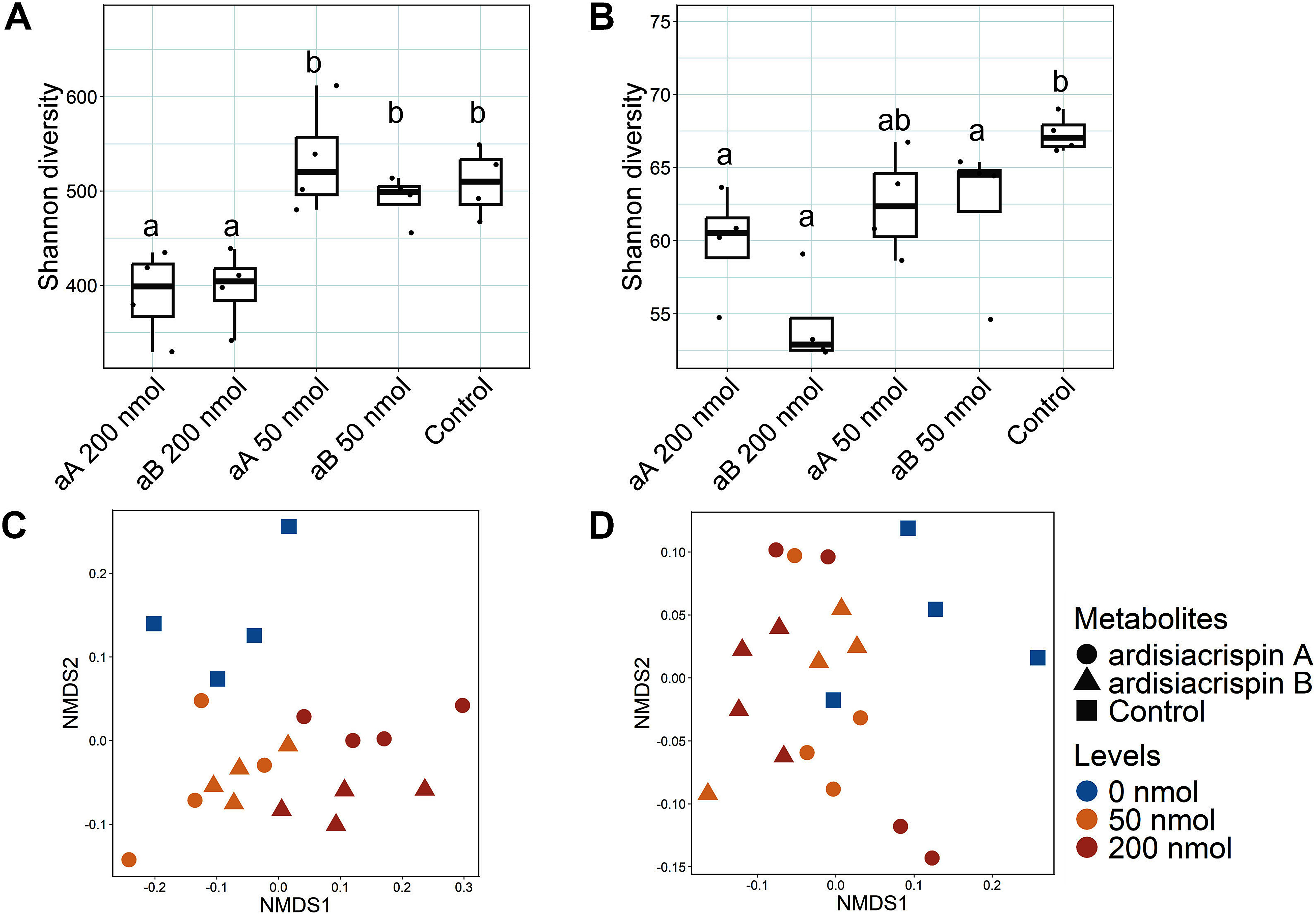
Figure 3. Shannon diversity of the bacterial (A) and fungal (B) communities in the forest soils (Kamigamo) treated with ardisiacrispin A (aA) and ardisiacrispin B (aB), 200 and 50 nmol g soil^−1^. Each control indicates untreated soil. Different letters (a–b) indicate statistically significant differences (*p*<0.05) by pairwise Wilcoxon test, corrected by the Bonferroni method. Nonmetric multidimensional scaling (NMDS) plots based on Bray–Curtis distances of bacterial (C) and fungal (D) communities in the soils treated with ardisiacrispin A (〇) and ardisiacrispin B (△), 200 (red), 50 (reddish brown) and 0 (blue) nmol g soil^−1^.

To investigate the effect of ardisiacrispin A and ardisiacrispin B on the soil microbiome, we treated forest soil (Kamigamo) with ardisiacrispins. For bacterial communities, treatments with ardisiacrispin A and ardisiacrispin B reduced Shannon diversity at a concentration of 200 nmol, but had no significant effect at 50 nmol (Figure 3A). NMDS analysis showed that the bacterial community composition varied depending on the saponin concentration (PerMANOVA, *p*<0.001), but was unaffected by the saponin type (i.e., ardisiacrispin A and B) (Figure 3C, Supplementary Table S2). For fungi, ardisiacrispin A significantly reduced the Shannon diversity at 200-nmol treatment, but not at 50-nmol treatment (Figure 3B). Ardisiacrispin B significantly reduced the Shannon diversity at both concentrations. NMDS analysis indicated that the fungal community composition varied depending on the saponin concentration (PerMANOVA, *p*=0.009), but was not significantly affected by the saponin type (Figure 3D; Supplementary Table S2).

**Figure 4:**
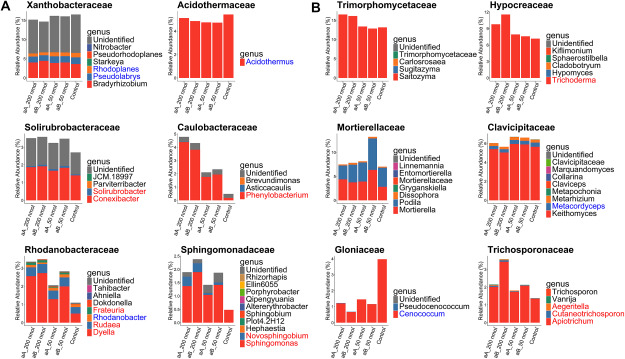
Figure 4. The mean relative abundances for the bacterial (A) and fungal (B) genera found in the top six families (excluding unidentified genera) in forest soil (Kamigamo) treated with ardisiacrispin A (aA) and ardisiacrispin B (aB), 200 and 50 nmol g soil^−1^. Red letters indicate genera that were significantly enriched, and blue letters indicate genera that were significantly depleted, in soils treated with 200 nmol or 50 nmol of saponins compared to the control (LEfSe; adjusted *p*-value<0.05).

As there was no difference in the effect of ardisiacrispin A and ardisiacrispin B on the bacterial and fungal community composition, the subsequent LefSe analysis was conducted without differentiating between the saponin types. For bacteria, LefSe analysis revealed that saponin treatment enriched 31 bacterial genera and depleted 37 genera in comparison with the control (Supplementary Figure S2A). The genera that were enriched by the saponin treatment included *Phenylobacterium* and *Novosphingobium* (Sphingomonadaceae), *Dyella* (Rhodanobacteraceae), and *Conexibacter* (Solirubrobacteraceae) (Figure 4A). In contrast, *Acidothermus* (Acidothermaceae) and *Candidatus Solibacter* (Rhodanobacteraceae) were among the genera that were depleted by the saponin treatment. For fungi, the saponin treatment enriched 13 fungal genera but depleted 14 genera when compared with the control (Supplementary Figure S2B). The genera that were enriched by the saponin treatment included *Trichoderma* (Hypocreaceae) and *Apiotrichum* (Trichosporonaceae) (Figure 4B). In contrast, *Cenococcum* (Gloniaceae) and *Metacordyceps* (Clavicipitaceae) were among the genera that were depleted by the saponin treatment. These results indicate that the saponin treatment leads to the enrichment of specific microbial species in forest soils and that this effect varies with the saponin concentration. We also evaluated the residual levels of ardisiacrispin A, ardisiacrispin B, and their aglycones in soil at the end of the 15-day incubation period, In both forest and field soils, ardisiacrispin A and ardisiacrispin B were completely degraded, and no clear peaks corresponding to aglycones were detected, indicating that both ardisiacripsins and their aglycones are not stable in the soil.

### Effect of ardisiacrispin treatment on the microbial communities in the field soil

**Figure 5:**
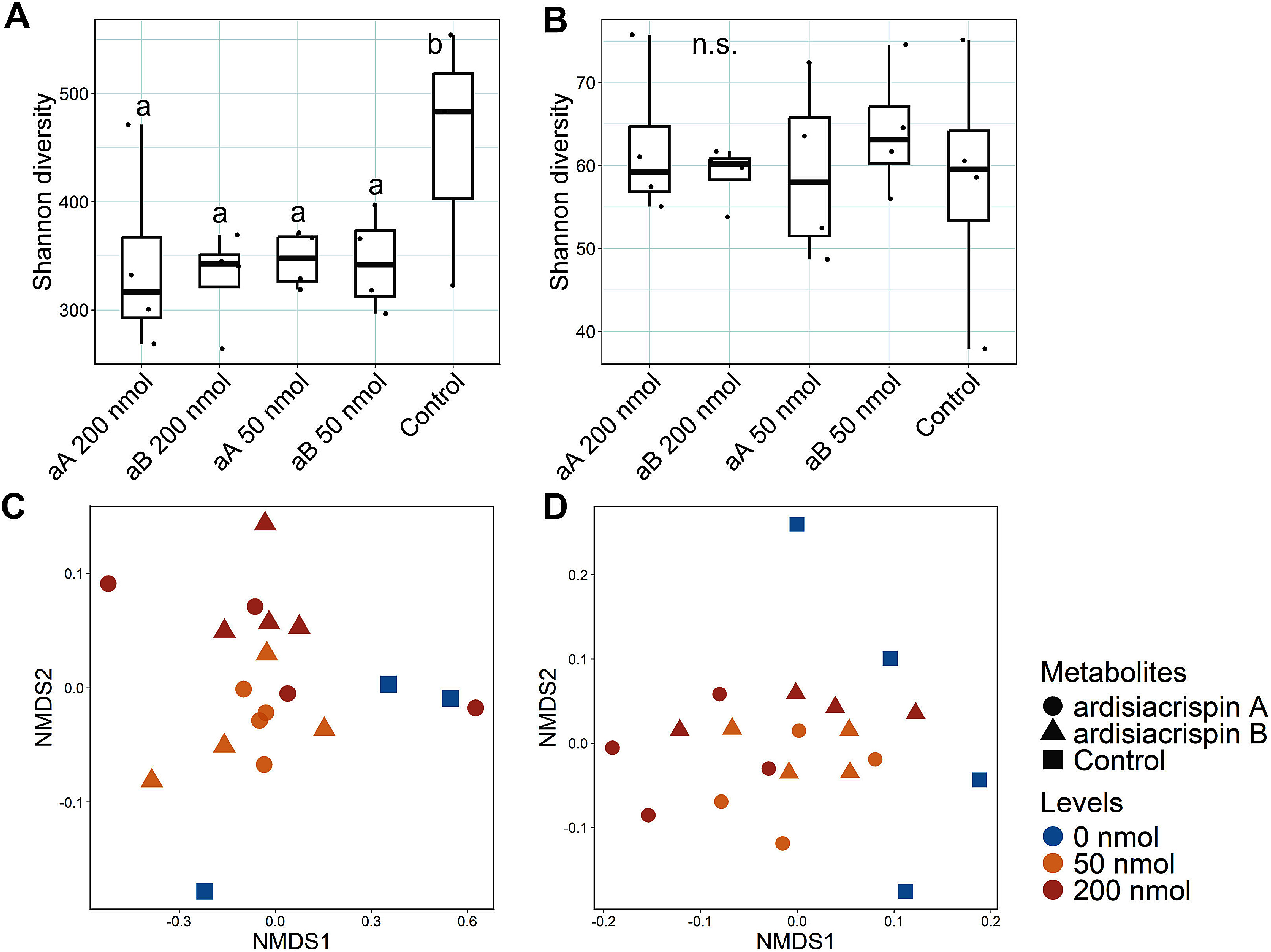
Figure 5. Shannon diversity of the bacterial (A) and fungal (B) communities in the field soils (Kameoka) treated with ardisiacrispin A (aA) and ardisiacrispin B (aB), 200 and 50 nmol g soil^−1^. Each control indicates untreated soil. Different letters (a–b) indicate statistically significant differences (*p*<0.05) by pairwise Wilcoxon test, corrected by the Bonferroni method, and n.s. indicates no significant differences. Nonmetric multidimensional scaling (NMDS) plots based on Bray–Curtis distances of bacterial (C) and fungal (D) communities in the soils treated with ardisiacrispin A (〇) and ardisiacrispin B (△), 200 (red), 50 (reddish brown) and 0 (blue) nmol g soil^−1^.

**Figure 6:**
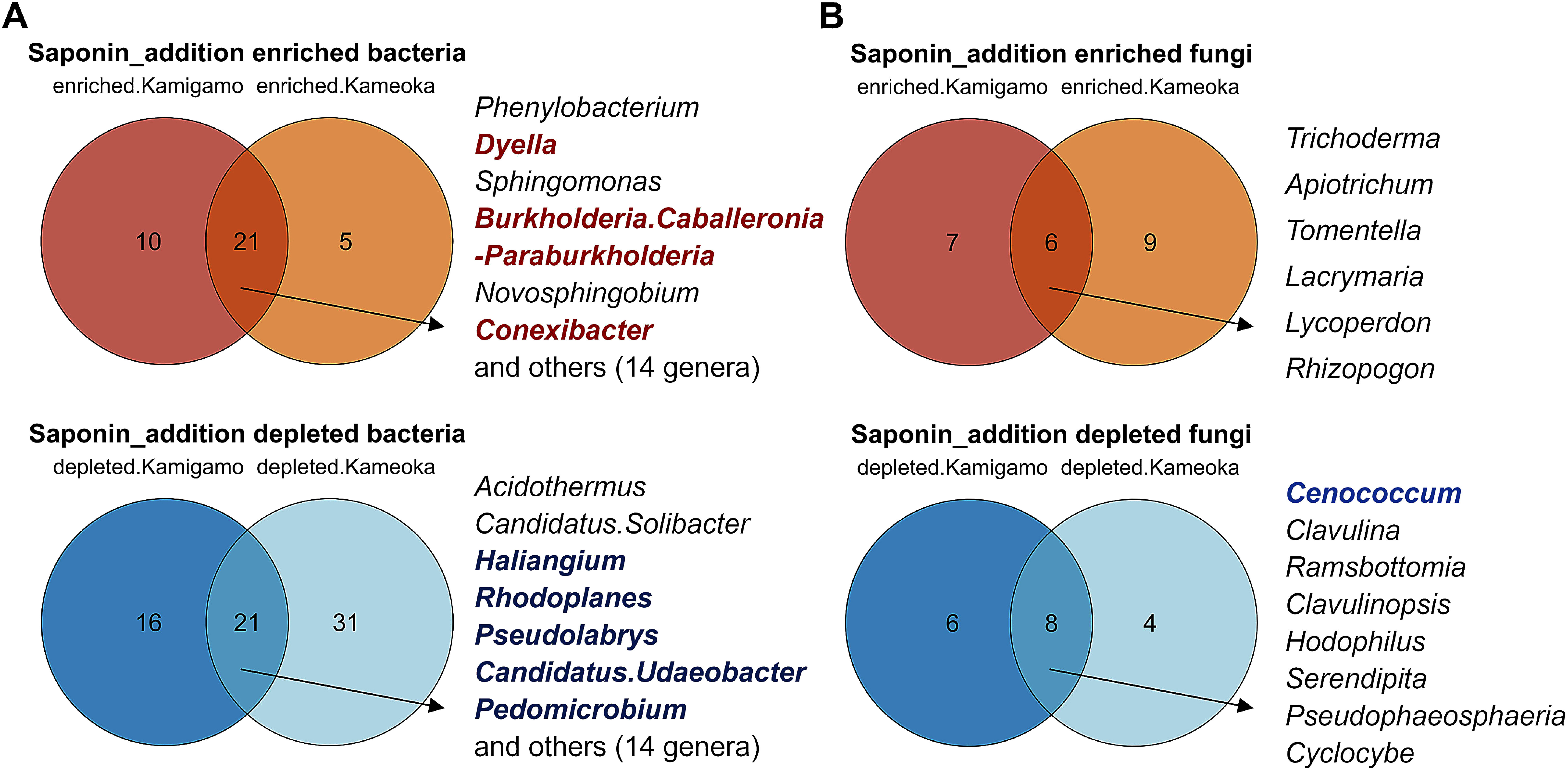
Figure 6. Venn diagrams illustrating the overlap of significantly enriched or depleted bacterial (A) and fungal (B) genera in forest soil (Kamigamo) and field soil (Kameoka) treated with ardisiacrispins. Genera enriched or depleted by saponin treatment represent those significantly enriched or depleted in soils treated with 200 nmol or 50 nmol of saponins compared to untreated control (LefSe; adjusted *p*-value<0.05). The genera commonly enriched or depleted are listed to the right of the diagram. Bold text indicates genera that were enriched (red) or depleted (blue) in the adult rhizosphere of *A. crenata*.

To assess whether the effects of saponins on microbial communities differed among the soil types, we conducted a saponin treatment experiment in field soil (Kameoka). The Shannon diversity of the bacterial communities decreased in the saponin-treated soil, whereas the fungal diversity was unaffected (Figure 5A, C). The microbial communities in the field soil and forest soil samples differed consistently, irrespective of the saponin treatment applied (Supplementary Figures S3, S4). The microbial community structure of both bacteria and fungi varied with the concentration of the applied saponin, but the difference was not significant by the saponin type (Figure 5B, D). Considering that there was no detectable variation in the way in which ardisiacrispin A and ardisiacrispin B influenced the microbial community structure, the subsequent LefSe analysis was conducted without differentiating between the saponin types. LEfSe analysis revealed that 21 bacterial genera were commonly enriched or depleted in saponin-treated soils across both forest soil and field soil (Figure 6A). In contrast, for fungi, six genera were commonly enriched and eight genera were commonly depleted in the saponin-treated soils across both the soil types (Figure 6B). These results suggested that the microbial species enriched or depleted by ardisiacrispins are, to some extent, shared across different soil types for both bacteria and fungi.

### Comparison of microbial taxa associated with ardisiacrispin and the rhizosphere of adult *A. crenata*

**Figure 7:**
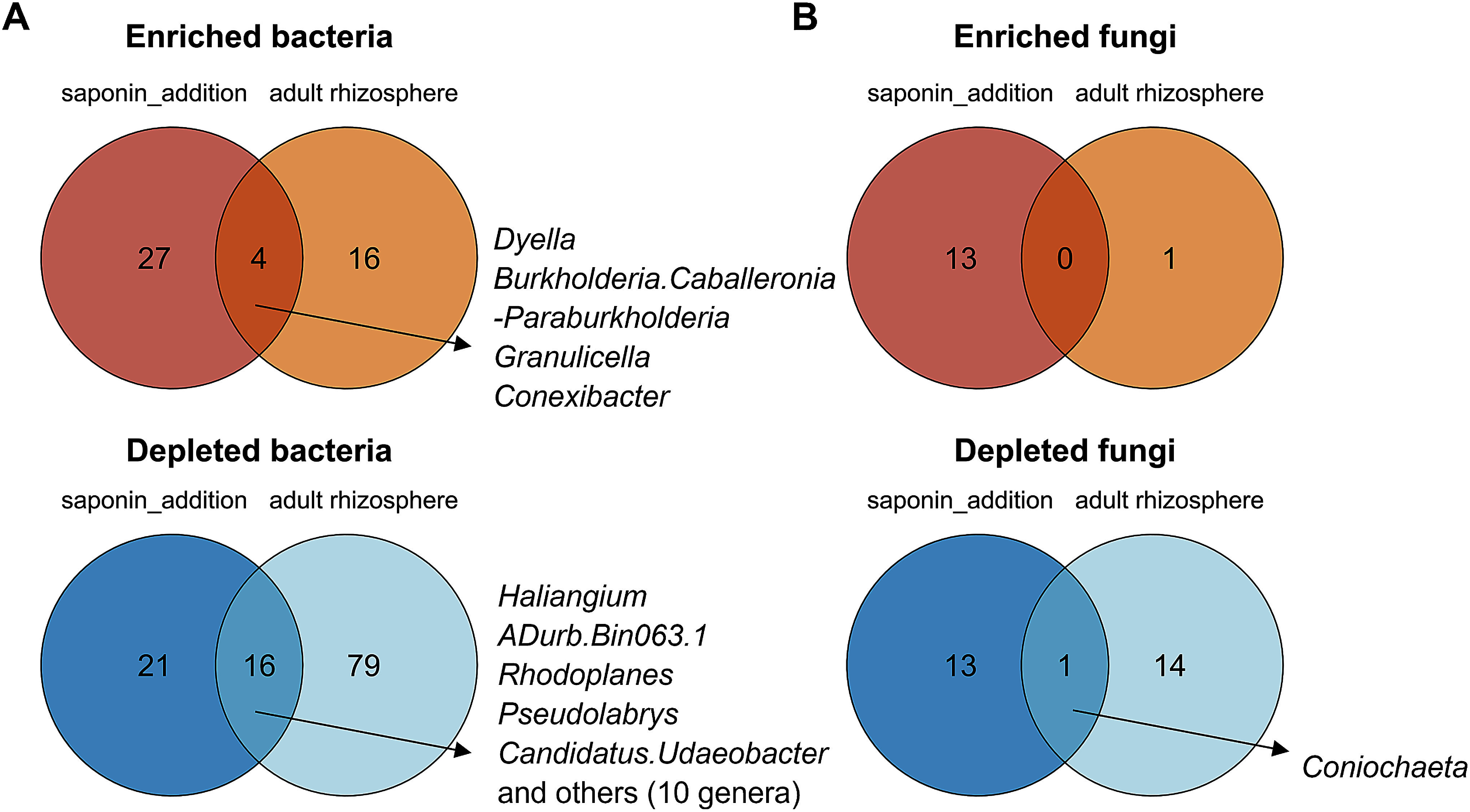
Figure 7. Venn diagrams illustrating the overlap of significantly enriched or depleted bacterial (A) and fungal (B) genera in forest soils treated with ardisiacrispins and in the adult rhizosphere of *A. crenata*. Genera enriched or depleted by saponin treatment represent those significantly enriched or depleted in soils treated with 200 nmol or 50 nmol of saponins compared to untreated control (LefSe; adjusted *p*-value<0.05). Genera enriched or depleted in the adult rhizosphere were identified by comparison with the seedling rhizosphere of *A. crenata* (LefSe; adjusted *p*-value<0.05). The genera commonly enriched or depleted are listed to the right of the diagram.

To further examine the effects of ardisiacrispins on the soil microbial composition, we examined the overlap between microbial genera affected by saponin treatment and those associated with the adult rhizosphere of *A. crenata*. Especially we focused on genera that were either enriched or depleted in both conditions. For bacteria, four genera, including *Dyella* and *Conexibacter*, were commonly enriched in both saponin-treated forest soil and the adult rhizosphere of *A. crenata* (Figure 7A). In contrast, 16 bacterial genera were commonly depleted under both conditions. For fungi, no genera were commonly enriched in both saponin-treated forest soil and the adult rhizosphere of *A. crenata*, whereas *Coniochaeta* was the only fungal genus that was commonly depleted under both conditions (Figure 7B). Overall, the differences in the microbial communities between the adult and seedling rhizospheres can be partially explained by the differences in their ardisiacrispin content, which indicates the effect of ardisiacrispins on the rhizosphere microbiome.

## Discussion

Plants can regulate rhizosphere microbial communities in accordance with their developmental stages (Chaparro et al. 2014; Che et al. 2024; Poppeliers et al. 2023; Sugiyama et al. 2014; Xiong et al. 2021). Previous studies have shown that certain PSMs can selectively enrich specific microbial taxa in the rhizosphere soil (Gfeller et al. 2023; Hu et al. 2018), although the mechanisms remain poorly understood. In this study, we combined field observations with the *in vitro* saponin treatment experiments to investigate the influence of saponins on the rhizosphere microbiome under natural ecosystems. Our results indicated that saponins accumulated in the rhizosphere soil attracted certain microbes in the natural ecosystems, suggesting the importance of saponin accumulation in plant-microbial interactions.

Both ardisiacrispin A and ardisiacrispin B were detected in the roots of the adult and seedling *A. crenata*. However, the rhizosphere soil of the adult individuals contained significantly higher concentrations of these saponins than that of seedlings, but the levels were barely detectable. These results indicate that, although ardisiacripsins are synthesized at both developmental stages, active exudation in the seedling stage is either absent or extremely low. Developmental-stage differences in the PSM secretion have been reported for grassland plants and crops, such as blueberry and soybean (Che et al. 2024; Csorba et al. 2022; Steinauer et al. 2023; Sugiyama et al. 2016). For example, Fujimatsu et al. (2020) showed that the accumulation of soybean-derived saponins increases with the developmental stage of soybean. Furthermore, their secretion has been observed to fluctuate in response to environmental factors (Korenblum et al. 2020). Considering that we collected adults and seedlings from the same site under comparable conditions, any environmental variation was minimized. Accordingly, we inferred that the observed difference in the rhizosphere saponin contents primarily reflects the developmental stage, highlighting the significance of developmental variation in the establishment of the rhizosphere microbial community through PSM exudation. Furthermore, because the roots of both adults and seedlings contained similar amounts of ardisiacrispins, it is likely that the adults actively secrete ardisiacrispins rather than lose them through passive leakage. Future studies will need to clarify how the differences in PSM profiles across developmental stages, as shown in this study, are related to the functional requirements at each developmental stage.

In the saponin-treatment experiments, the effects of ardisiacrispin A and ardisiacrispin B on the soil microbiome did not differ significantly. These two saponins differ only in their terminal sugar moieties (Figure 1A); however, whether this structural difference affects their degradation pathways in the soil remains unclear. Zhou et al. (2023) found that ardisiacrispin B undergoes hydrolysis in rat plasma to produce an aglycone. While the catabolic pathway differs from those in the rat plasma, both ardisiacrispin A and B could generate the same aglycone by hydrolysis in the soil, which potentially explains the observed similarity in the soil microbial response. In fact, past studies on tomato- and alfalfa-derived saponins have exhibited that they are broken down by soil microbes into aglycones, which subsequently interact with soil microbes (Nakayasu et al. 2021a; Takamatsu et al. 2023; Tava et al. 2025). This finding supports the possibility that ardisiacrispin A and ardisiacrispin B follow a similar degradation pathway, yielding comparable effects on the soil microbiome. In contrast, Li et al. (2020) found that ginseng-derived saponins with different sugar moieties (i.e., ginsenoside Rb1, Rh1, and Rg1, all of which are composed of the same aglycone) enriched distinct fungal genera in the soil. Although our study did not detect microbial community shifts linked to sugar moiety variation, the observed decrease in the fungal diversity by saponin treatment in forest soil (Figure 3B) may imply that fungi are more responsive to sugar moiety differences than bacteria.

The saponin-treatment experiment revealed that ardisiacrispins have the potential to mediate the assembly of soil bacterial and fungal communities in both forest and field soil samples. For bacteria, *Novosphingobium* and *Phenylobacterium* were enriched in both forest and field soils, which is consistent with their enrichment in previous soybean- and licorice-derived saponin treatments (Fujimatsu et al. 2020; Nakayasu et al. 2021b), suggesting that these genera respond broadly to triterpenoid saponins irrespective of their structural differences. In contrast, *Conexibacter* and *Dyella*, which also showed enrichment in both forest and field soils under ardisiacrispin supplementation, have not been reported to respond similarly to soybean- and licorice-derived saponins, implying that ardisiacrispin uniquely recruits certain microbial taxa across soil types. These patterns suggest that ardisiacrispin enriches soil microbes commonly associated with other saponins and selectively enriches ardisiacrispin-specific microbial taxa across soil types. However, Cadot et al. (2021) grew wild-type maize and benzoxazinoid-deficient mutants in various soil types and demonstrated that the bacterial taxa enriched by benzoxazinoids differ with the soil type. The consistent saponin effect on the soil microbiomes observed in this study may reflect our *in vitro* approach, which minimized environmental and host-plant factors. Future studies should be conducted under a broad spectrum of field conditions to further refine our understanding of how saponin influences the shape of soil-microbial assemblages across ecological gradients. Fungal communities also respond to saponins, with *Trichoderma* showing enrichment reminiscent of patterns reported for ginsenoside-derived saponins (Li et al. 2020). A species of *Trichoderma* is known for its growth-promotion effects on a wide range of hosts, such as tomato and brassica plants (Toju et al. 2020), which is achieved through their antifungal activity and the induction of a systemic-acquired resistance (SAR)-like response, which directly promotes plant growth (Dutta et al. 2023; Studholme et al. 2013; Vinale et al. 2008). However, it remains unclear whether *A. crenata* and *Trichoderma* spp. interact directly, indicating the need for additional investigation. Overall, these findings underscore the importance of examining the influence of saponin under diverse conditions to clarify how these bioactive metabolites drive PSM-microbe interactions.

Among the potential mediators of plant-microbe interaction in rhizosphere soil, saponins have emerged as a plausible factor considering that they exhibit significantly higher concentrations in the rhizosphere of adult *A. crenata* relative to that in the rhizosphere of seedlings, and several bacterial genera consistently enriched or depleted in saponin-treated forest soil, mirroring the observed shifts from seedling to the adult rhizosphere. Although other root exudate components, such as fatty acids, amino acids, and sugars, also affect microbial communities (Badri et al. 2009; Moe 2013; Wen et al. 2021), our observations suggest that saponin exudation can selectively alter the microbial composition of soil in the natural ecosystem. Further investigation into how saponin-enriched microbes influence both the surrounding microbial communities and host-plant growth is expected to provide valuable insights into the complex feedback mechanisms that govern plant-microbe interactions (Foster et al. 2017). Previous studies on soybean, tomato, and tobacco have identified the catabolic pathways and genes involved in the degradation of PSMs by focusing on the soil microbes enriched by these compounds (Aoki et al. 2024; Nakayasu et al. 2023; Shimasaki et al. 2021). Similarly, *Conexibacter*, *Dyella*, *Granulicella*, *Burkholderia*, *Caballeronia*, and *Paraburkholderia*, which were consistently enriched in the rhizosphere of adult *A. crenata* as well as in response to ardisiacrispin treatment, may be involved in the metabolism of ardisiacrispins in the rhizosphere of *A. crenata*. Nevertheless, the saponin-treatment experiment did not fully replicate the fungal community composition of adult rhizospheres, possibly because fungi exhibit greater sensitivity to competition and environmental fluctuations (He et al. 2023), suggesting that the additional biotic or abiotic factors may mask the saponin effects.

In conclusion, although several studies have investigated the interaction between PSMs and rhizosphere soil microbes in agricultural or field soils, our findings suggest that PSMs are also exuded from the roots, thereby exerting selective pressure on the rhizosphere microbial communities in the natural ecosystems. In the rhizosphere of *A. crenata*, a moderate number of bacterial taxa were estimated to be either enriched or depleted via saponin exudation. Although this study did not directly evaluate the host–microbe interactions, we identified candidate microbial taxa that may have ecologically significant interactions with ardisiacrispins. Future research should employ soil transplantation experiments with saponin-treated soil within the natural habitat of *A. crenata* so as to determine whether these microbes influence the host fitness, population dynamics, and broader ecosystem functions.

## Abbreviations

ASVs: amplicon sequence variants
LC-MS: liquid chromatography-mass spectrometry
LDA: linear discriminant analysis
LefSe: linear discriminant analysis effect size
NMDS: non-metric multidimensional scaling
OTUs: operational taxonomic units
PerMANOVA: permutational multivariate analysis of variance
PSMs: plant specialized metabolites
UPLC: ultra-performance liquid chromatography

## Description of supplementary files

Supplementary Table S1. PerMANOVA results for the three soil compartments associated with *A. crenata*.

Supplementary Table S2. PerMANOVA results for the saponin treatment experiment.

Supplementary Figure S1. Photographs of seedlings and adult *Ardisia crenata*.

Supplementary Figure S2. Venn diagrams illustrating the overlap of significantly enriched or depleted genera in the saponin treatment experiment (forest soil).

Supplementary Figure S3. The genus-level taxonomic composition in saponin treatment experiments (bacteria).

Supplementary Figure S4. The genus-level taxonomic composition in saponin treatment experiments (fungi).
