# Supplementary material for: Developmental stage-specific triterpenoid saponin accumulations in Ardisia crenata rhizosphere and its influence on rhizosphere microbial communities
Source: Plant Biotechnol (Tokyo). 2025 Sep 25;42(3):357–69. doi: 10.5511/plantbiotechnology.25.0504a (PMC12573610; doi:10.5511/plantbiotechnology.25.0504a)
Supplement: Supplementary Data [file plantbiotechnology-42-3-25.0504a_s001.pdf]

Supplementary materials

Supplementary Table S1

Microbial community difference among the three soil compartments associated with *A. crenata* (adult rhizosphere, seedling rhizosphere soil and bulk soil) tested by PerMANOVA. The results of pairwise comparisons are shown, with p-values adjusted.

Variables				PerMANOVA results		
				df	*F*	*P*
Bacteria	bulk soil	vs.	seedling rhizosphere	1	**3.95**	**0.024**
	bulk soil	vs.	adult rhizosphere	1	**6.72**	**0.0228**
	adult rhizosphere	vs.	seedling rhizosphere	1	**5.90**	**0.0249**
				1		
Fungi	bulk soil	vs.	seedling rhizosphere	1	**2.85**	**0.024**
	bulk soil	vs.	adult rhizosphere	1	**2.64**	**0.0228**
	adult rhizosphere	vs.	seedling rhizosphere	1	1.00	1

Notes : Significant terms ($P < 0.05$) are shown in bold.

Supplementary Table S2

The results of PerMANOVA for fungal and bacteria community compositions to test the difference among the three saponin concentrations (control, 50 nmol g^{-1} and 200 nmol g^{-1}) and type of saponin (ardisiacrispin A and ardisiacrispin B).

Soil types	Targets	Variables	Df	*F*	R^2	*P*
Kamigamo (forest soil)	Bacteria	Levels	2	4.27	0.32869	**0.001**
		Metabolites	1	1.34	0.0515	0.136
		Levels: Metabolites	1	1.10	0.04217	0.269
	Fungi	Levels	2	2.23	0.20872	**0.009**
		Metabolites	1	1.30	0.06087	0.191
		Levels: Metabolites	1	0.63	0.02934	0.817
Kameoka (field soil)	Bacteria	Levels	2	3.33	0.29753	**0.001**
		Metabolites	1	0.84	0.0377	0.667
		Levels: Metabolites	1	0.90	0.04006	0.561
	Fungi	Levels	2	1.75	0.17436	**0.001**
		Metabolites	1	0.90	0.04474	0.638
		Levels: Metabolites	1	0.72	0.03559	0.938

Notes : Significant terms ($P < 0.05$) are shown in bold.

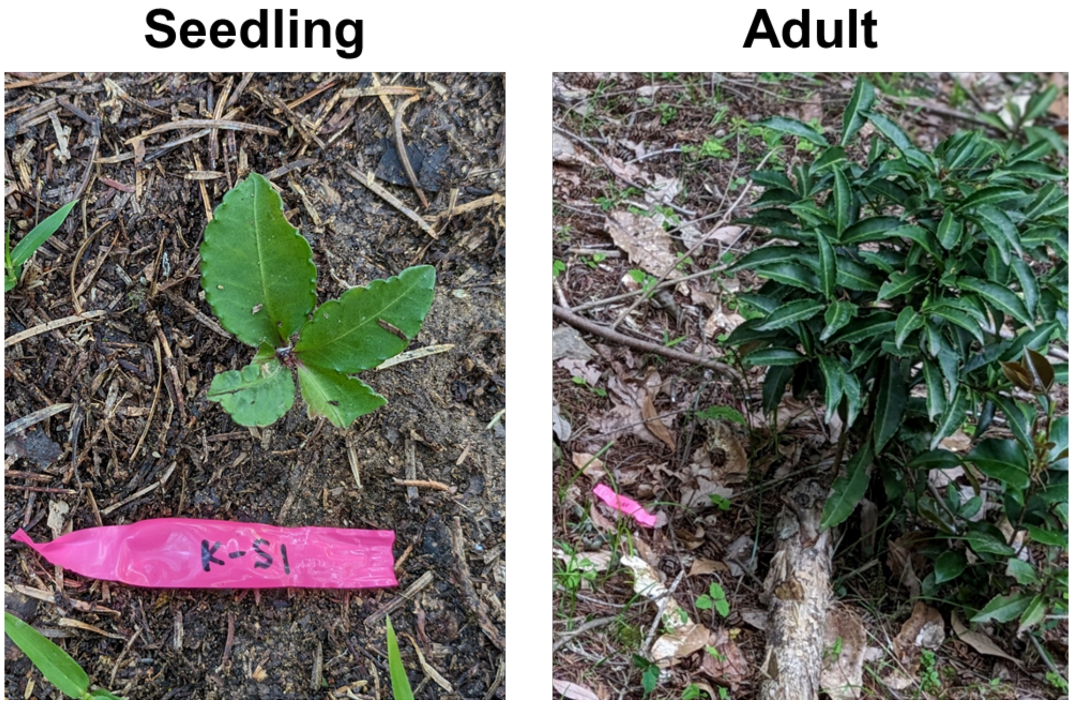

Supplementary Figure S1

Photographs of *Ardisia crenata* seedlings(height ≤ 10 cm) and adult (height ≥ 20 cm) sampled at the Kamigamo Research Station, Kyoto University. The vertical width of the pink tapes visible in the photographs are 15 mm.

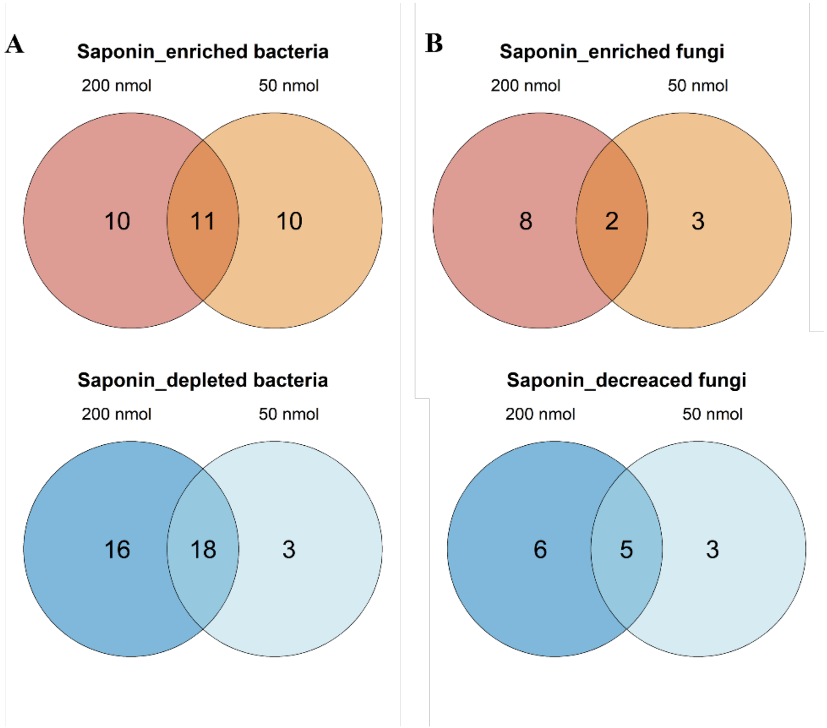

Supplementary Figure S2

Venn diagrams showing the overlap of significantly enriched or depleted bacterial (A) and fungal (B) genera in forest soil (Kamigamo) treated with saponins at 50 nmol g^{-1} and 200 nmol g^{-1}.

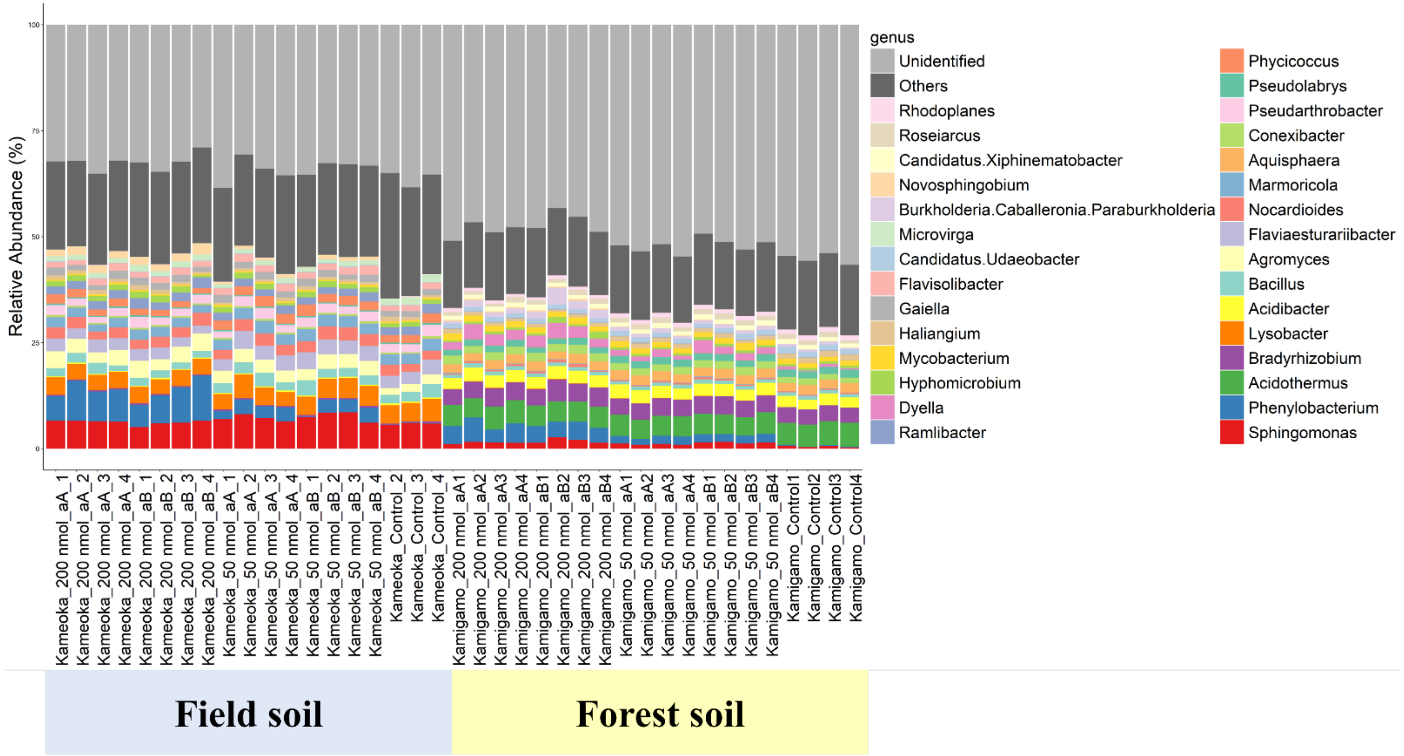

Supplementary Figure S3

The genus-level taxonomic composition of soil bacterial community in forest soil (Kamigamo) and field soil (Kameoka) treated with ardisiacrispin A (aA) and ardisiacrispin B (aB). The top 30 taxa were displayed. “Kameoka_control_1” was excluded from the analysis due to a technical issue—specifically, a low number of sequencing reads. All remaining species are consolidated into the 'Others' category. The vertical axis indicates the relative abundance of each taxon.

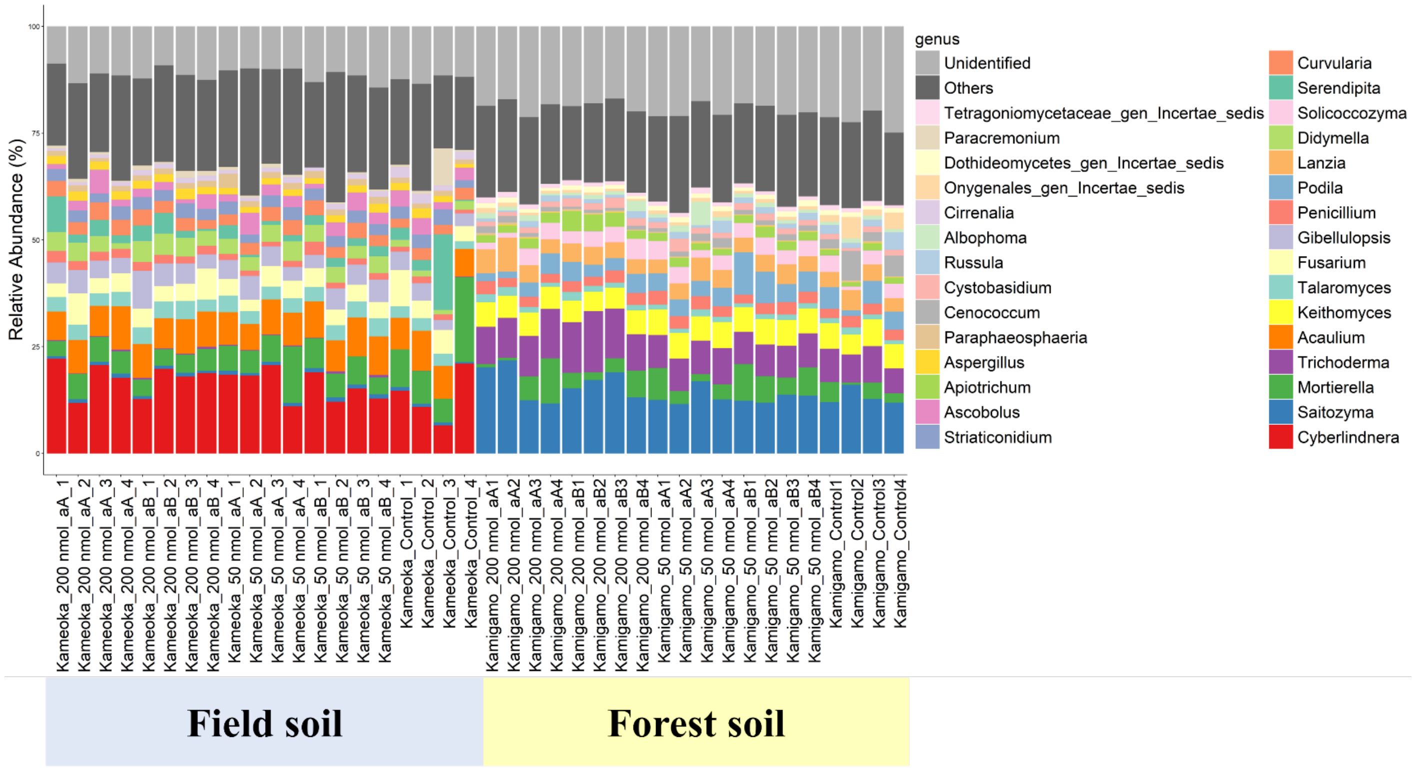

Supplementary Figure S4

The genus-level taxonomic composition of soil fungal community in forest soil (Kamigamo) and field soil (Kameoka) treated with ardisiacrispin A (aA) and ardisiacrispin B (aB). The top 30 taxa were displayed. All remaining species are consolidated into the 'Others' category. The vertical axis indicates the relative abundance of each taxon.
